# The value of error-correcting responses for cognitive assessment in games

**DOI:** 10.1038/s41598-024-71762-z

**Published:** 2024-09-04

**Authors:** Benny Markovitch, Nathan J. Evans, Max V. Birk

**Affiliations:** 1 Human Technology Interaction, Eindhoven University of Technology, 5612 Eindhoven, AZ The Netherlands; 2 Department of Psychology, Ludwig Maximilian University of Munich, 80799 Munich, Germany; 3 School of Psychology, University of Queensland, St Lucia, 4067 Australia

**Keywords:** Game-based cognitive assessment, Interference control, Response inhibition, Response-rule switching, Change of mind, Error-correction, Human behaviour, Diagnostic markers

## Abstract

Traditional conflict-based cognitive assessment tools are highly behaviorally restrictive, which prevents them from capturing the dynamic nature of human cognition, such as the tendency to make error-correcting responses. The cognitive game Tunnel Runner measures interference control, response inhibition, and response-rule switching in a less restrictive manner than traditional cognitive assessment tools by giving players movement control after an initial response and encouraging error-correcting responses. Nevertheless, error-correcting responses remain unused due to a limited understanding of what they measure and how to use them. To facilitate the use of error-correcting responses to measure and understand human cognition, we developed theoretically-grounded measures of error-correcting responses in Tunnel Runner and assessed whether they reflected the same cognitive functions measured via initial responses. Furthermore, we evaluated the measurement potential of error-correcting responses. We found that initial and error-correcting responses similarly reflected players’ response inhibition and interference control, but not their response-rule switching. Furthermore, combining the two response types increased the reliability of interference control and response inhibition measurements. Lastly, error-correcting responses showed the potential to measure response inhibition on their own. Our results pave the way toward understanding and using post-decision change of mind data for cognitive measurement and other research and application contexts.

## Introduction

The behaviorally restrictive design of typical cognitive assessment tools has been criticized for failing to capture the dynamic nature of human cognition and decision-making^1–4^, including common behaviors such as error-corrections^5–7^. This is because most cognitive assessment tools are too behaviorally restrictive to allow error-correcting responses to follow mistaken initial responses in the same trial^4–7^. This limitation also applies to most cognitive games, which use game-like environments to assess cognitive functions, yet are typically as behaviorally restrictive as other cognitive assessment tools^4,8–14^. Critiques of the overly restrictive designs of cognitive assessment tools motivated the development of a less restrictive cognitive game called Tunnel Runner^4^. Tunnel Runner gives players continuous control over their interaction with the cognitive assessment system and provides players with natural opportunities and incentives to correct mistaken initial responses^4^. However, since error-correcting responses are rarely considered in cognitive assessment, the psychometric and theoretical foundations needed to use error-correcting responses are lacking, meaning that error-correcting responses remain unused even when allowed, encouraged, and measured^4^. Crucially, this limits the potential benefits of cognitive games, such as Tunnel Runner, which tend to evoke relatively high rates of mistaken initial responses^4,13^ that are ignored by reaction-time-based measurements, leading to increased data loss and reduced measurement efficiency. However, since error-correcting responses can reflect the same cognitive functions measured with initial response data^5,15^ and could create new cognitive measurement opportunities^5–7^, they might be a valuable source of cognitive data.

Historically, cognitive tasks were designed to evoke repeatable experimental effects, rather than reliably assess individual differences^16^. This is reflected in the reliance on conflict effects, which compare performance on regular trials against conflict trials that place additional demands on specific cognitive functions, such as response inhibition in stop-signal tasks^17,18^, and interference control in flanker tasks^19^. Conflict tasks’ psychometric reliability, defined by the ratio between individual differences and measurement error^20^ is limited by the tasks’ reliance on reaction time (RT) differences between trial types^20,21^, and their tendency to be demotivating and disengaging^4^ which can lead people to respond inconsistently and fail to perform to their full ability. These and other factors^20^ lead many conflict-based tasks to exhibit unacceptably low reliability^16^. However, due to the considerable interest in using conflict-based measurements in scientific research^20,22–27^ and health care^28,29^, several approaches have been proposed to improve their reliability. These proposals include using theoretically-informed response models^30^, evoking stronger conflict effects^31^, and providing better experiences via cognitive games^4,8,14^. These different approaches can be complemented by the use of error-correction data, which may increase the amount of response data, and could provide new opportunities to measure cognition.

Using error-correcting responses for cognitive assessment requires a theoretically-grounded understanding of the cognitive functions involved and how they may relate to the cognitive functions measured by correct initial responses. Models of cognition involving processes such as evidence accumulation in favor of specific decisions^7,32^, or competition between response inhibition and initiation processes^17,18^, have traditionally been developed to understand initial response data. However, evidence accumulation models have shown the ability to account for error-correcting responses by assuming that error-correcting responses reflect a continuation of the evidence accumulation process after initial responses^5,33^. These findings converge with the observation that responses in trials following erroneous responses are faster when they match what would have been error-correcting responses^34,35^ to support the hypothesis of cognitive continuity. According to the hypothesis of cognitive continuity (or spillover^5^), error-correcting responses reflect instances where the cognitive processes responsible for initial responses continue their operation after erroneous initial responses were made to later evoke error-correcting responses in the same trial. By outlining how error-correcting responses relate to the cognitive functions measured with correct first responses, cognitive continuity provides a path toward understanding and using error-correcting responses.

Cognitive continuity implies that error-correcting responses can be used for cognitive assessment alongside correct initial responses because error-correcting responses reflect a continuation of the processes measured by the initial responses^5^. However, the extent of cognitive continuity is contested^33^, and continuity between correct initial and error-correcting responses has not been directly examined in conflict-based measurements, whose use of difference scores may limit cognitive continuity as continuity between response types per each trial type does not guarantee continuity in the differences between conflict and non-conflict trials. However, if cognitive continuity holds with conflict-based measurements, then error-correcting responses could be used to supplement or even replace first-response-based measurements, as both response types would reflect the same cognitive functions. This could allow error-correcting responses to become an important part of cognitive assessment.

Although cognitive continuity may resolve theoretical challenges to the use of error-correcting responses in conflict-based measurements, psychometric challenges remain. Specifically, statistical approaches for the use of error-correcting responses in conflict-based cognitive assessment have, to our knowledge, been neither developed nor evaluated. This is a problem, because the estimation of individual differences in cognitive functions using initial responses alongside error-correcting responses can be complex. Specifically, the statistical estimation method should reflect both continuity and differences between the two response types across different trial types^33^. However, since this type of assessment has not been performed with conflict-based measures, it is unclear how error-correcting responses can be used for conflict-based cognitive assessment.

In this article, we address key theoretical and psychometric challenges to the use of error-correcting responses in conflict-based cognitive assessment using behavioral data collected via Tunnel Runner. Tunnel Runner includes conflict-based measures of interference control, response inhibition, and response-rule switching^4^, which are used to limit the influence of irrelevant stimuli on behavior^22^, suppress dominant but inappropriate behavioral tendencies^22^, and flexibly adapt to changing environments^22,36^, respectively. Crucially, Tunnel Runner’s continuous player control allows and encourages players to make error-correcting responses in trials where they made incorrect initial responses^4^; as unlike typical cognitive measurement tools^5,6^, Tunnel Runner’s trials do not terminate immediately after an initial response. This feature uniquely enables Tunnel Runner to naturally encourage and measure players’ error-correcting responses, and makes it a unique research paradigm to assess cognitive continuity with different conflict effects and to develop and evaluate statistical approaches to use error-correcting responses for conflict-based cognitive measurement. For these reasons, we used behavioral data collected through Tunnel Runner to assess the potential value of error-correcting responses for cognitive assessment in conflict-based tasks. Specifically, we sought to answer the following interconnected research questions:

RQ1: Do error-correcting responses show cognitive continuity with initial responses in conflict-based cognitive measurements?
RQ2: Can error-correcting responses be used alongside correct initial responses to increase the reliability of conflict-based cognitive measurements?
RQ3: Can error-correcting responses be used as cognitive measurements on their own?

## Material and methods

### Tunnel runner

Tunnel Runner^4^ is an infinite runner game in which a group of five player-controlled rats run through a tunnel filled with obstacles, with different aspects of the game designed to create response conflicts to assess players’ interference control, response inhibition, or response-rule switching. We focus on describing the relevant gameplay sections and cognitive measurements, as a full description and validation of the game has been published elsewhere^4^.

**Fig. 1:**
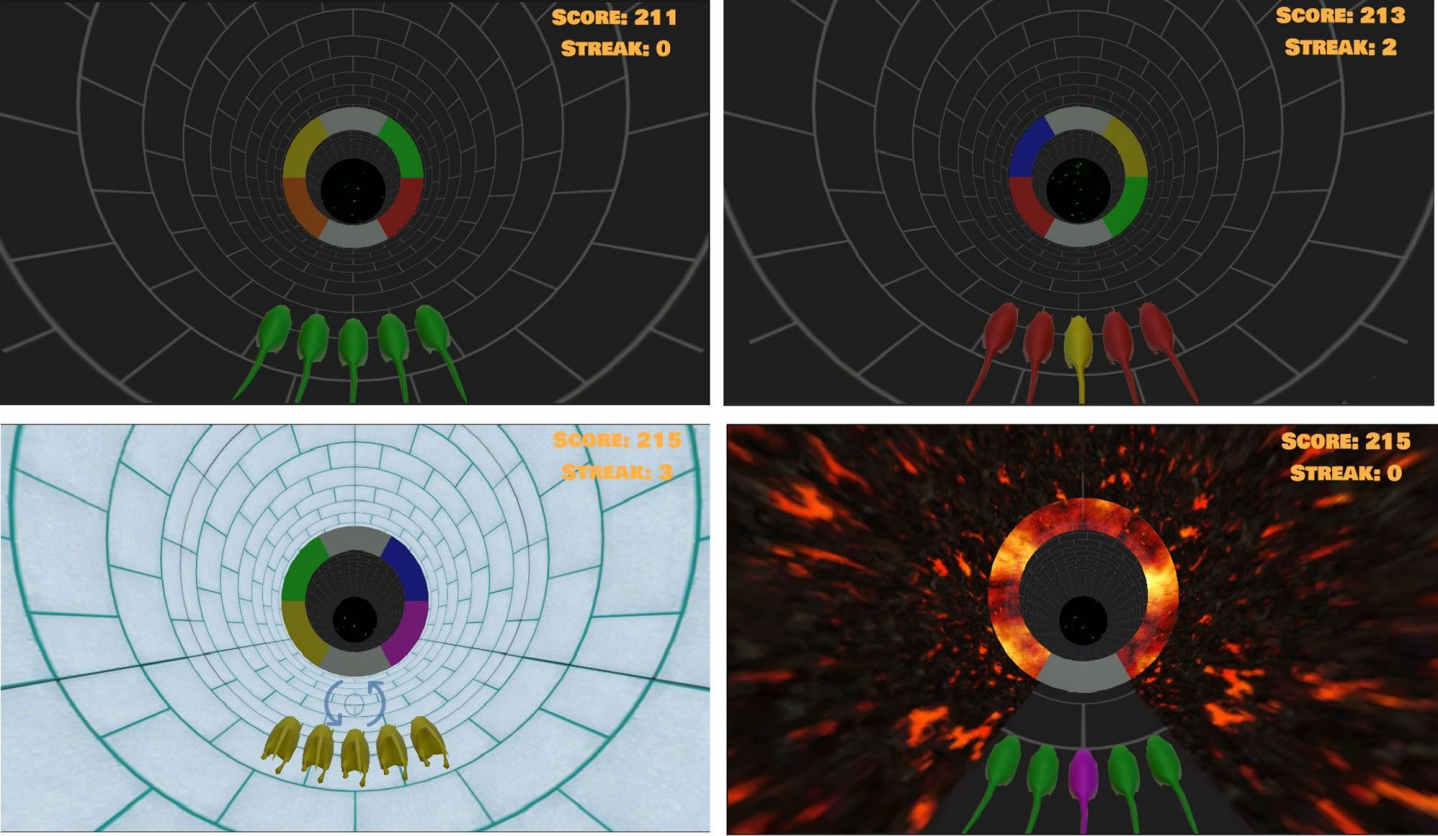
Tunnel Runner’s trial types. In regular trials (top left), all rats have matching color. In mismatching flanker trials (top right), the flanker rats are differently colored from the central rat. In ice trials (bottom left), the relation between player input and the rats’ movement is reversed. In lava trials (bottom right), a delayed stop-signal appears in the form of lava, penalizing players for moving the rats.

The goal of the player is to guide the central rat to the section of the circular obstacle upcoming in the tunnel that matches the central rat’s color, requiring the player to rotate the rats’ positioning (Fig. 1). As the rats move through the tunnel, players can hold the A key to rotate the rats to the left and the L key to rotate them to the right, which also allows players to change directions and correct mistaken initial responses. The rotation is continuous rather than ballistic; only lasting while players hold down a direction key. Good performance is motivated by rewarding players with points equivalent to the number of times in a row (the streak) that the central rat passed through the correct section of the obstacle. After passing through an obstacle, all the rats are colored gray and their rotation is disabled. New colors are then assigned to the central and flanking rats, 433 and 350 milliseconds, respectively, after the new obstacle is presented. Rotation is re-enabled once the central rat’s color was assigned.

Tunnel Runner starts with 40 training trials, which are not used for assessment. These are followed by 126 regular trials, 126 mismatching flanker trials, 84 lava trials, and 120 ice trials. There are 2 breaks after the training trials, dividing the test trials into 3 blocks.

#### Regular trials

In 126 regular (congruent flanker) trials, shown at the top left of Fig. 1, the central rat and the four flanking rats are assigned the same color after the obstacle is presented. The rats can be rotated after the central rat’s color assignment, leaving players with 1317 ms to pass through the correct section of the obstacle before the next trial begins. The time until obstacle collision is adapted based on the outcomes of regular and mismatching flanker trials. Passing through the correct section reduces the time between the next color assignment and obstacle collision, whereas passing through the incorrect section increases the time between color assignment and obstacle collision. This adaptation targets success rates of 80%.

#### Mismatching flanker trials

In 126 regular mismatching flanker trials, depicted on the upper right of Fig. 1, the flanker rats are assigned the color of the section opposite to the correct section. Since flanker rats match the correct section in 50% of trials, players are asked to ignore them. These trials enable the measurement of players’ capacity for interference control^4^ via the differences between RTs on matching and mismatching flanker trials.

#### Lava trials

During 84 lava trials, which measure players’ response inhibition^4^ and are depicted at the bottom right of Fig.  1, the rats are surrounded by lava (a stop-signal) after color assignment. Touching the lava, which can only be prevented by not moving the rats, leads players to continuously lose points until they rotate the rats back to the starting point. Lava trials are independent of the flanker condition, such that the colors of the central and flanker rats are equally likely to match or mismatch. Creating 42 matching and 42 mismatching flanker lava trials. At first, the lava appears 300 milliseconds after the central rat is assigned a color. The stop-signal delay is then adapted based on the player’s performance, and the delay in matching flanker trials is adapted independently of the delay in mismatching flanker trials. Moving the rats after the stop-signal results in lava appearing 50 milliseconds earlier, giving players more time to stop early. Successful inhibition causes lava to appear 50 milliseconds later, giving players less time to stop early. This adaptation leads players to inhibit around 50% of responses in lava trials^4^, which is needed to calculate the stop-signal reaction time measure of response inhibition by the integration method^17,18^.

#### Ice trials

During 120 ice trials, depicted on the bottom left of Figure 1, the tunnel is filled with ice as soon as the rats pass the previous obstacle, reversing the key mapping so that holding the A key rotates the rats to the right, while the L key rotates them to the left. Matching and mismatching flanker trials are equally spread across ice trials, while fire trials rarely overlap with ice trials as this combination is not used for measurement. Ice trials enable the measurement of players’ capacity for response-rule switching via the differences between RT in ice and non-ice trials^4^.

### Operationalizing error-correcting responses in tunnel runner

We aimed to define and measure error-correcting responses according to leading models of speeded binary decision-making and response inhibition. Importantly, as Tunnel Runner enables different types of error-correcting behaviors, such as response-switching in non-lava trials and late stopping in lava trials, and these different behaviors are typically modeled using different frameworks, we considered distinct theoretical perspectives for each type of error-correcting behavior. Specifically, we considered error-correcting responses that involve response-switching to result from an evidence accumulation process^5,15,32,37^, which is the dominant perspective on speeded binary decisions^7^. In contrast, we considered error-correcting responses involving late stopping in lava trials to result from a competition between two independent cognitive processes^17^, which is the dominant perspective on response inhibition in the stop-signal literature^18^.

#### Error-correction in non-lava trials

To operationalize error-correcting responses in non-lava trials, we assumed that the timing and nature of players’ initial and error-correcting responses depend on the dynamics of an evidence accumulation process. The evidence accumulation perspective^7,32^ involves the accumulation of evidence until it reaches a response boundary for one of the competing decisions. Once enough evidence has accumulated to reach a response boundary, the corresponding response is initiated.

**Fig. 2:**
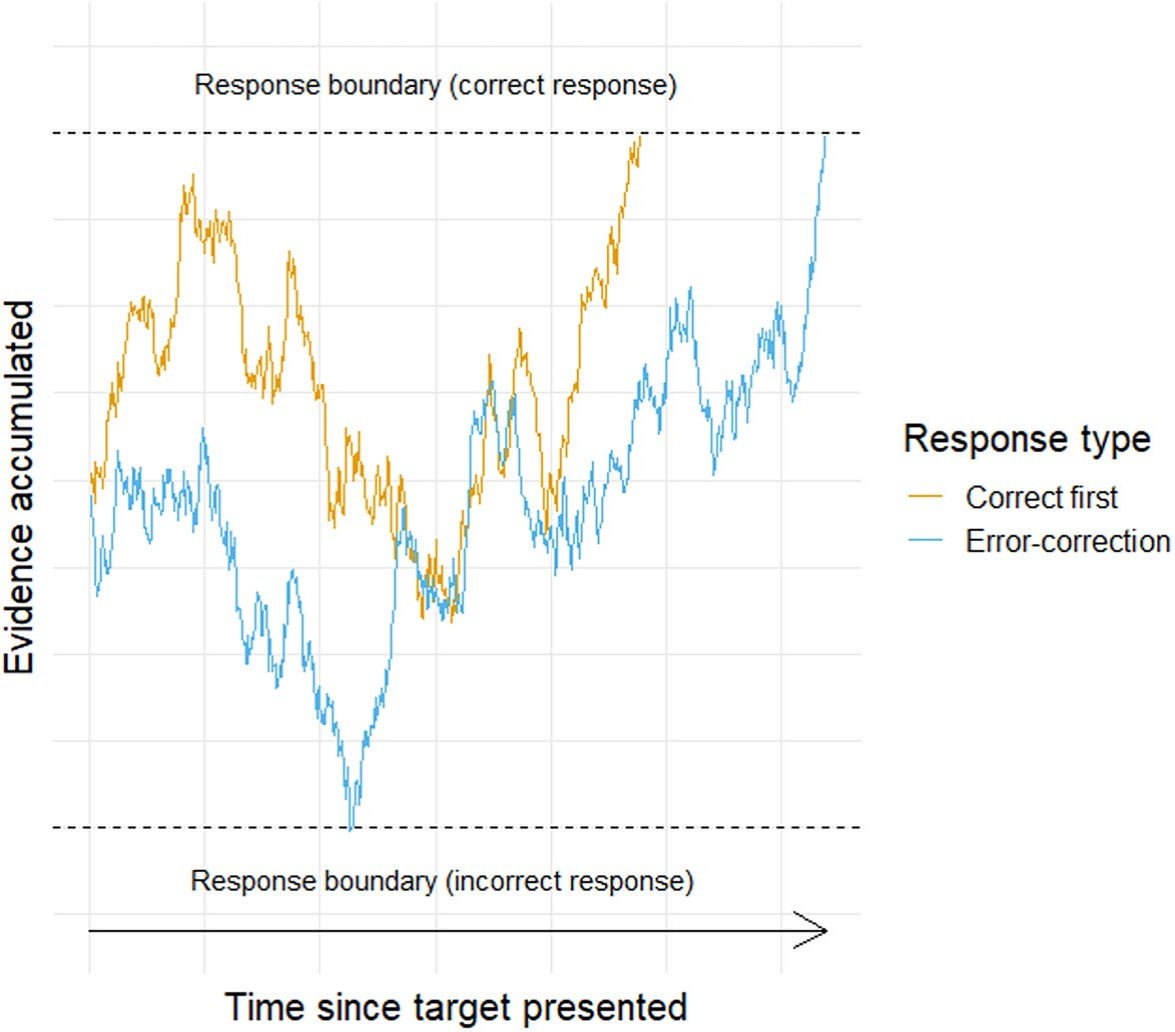
A simplified evidence accumulation process representation of two response types. For the correct first response, evidence accumulates to evoke a correct first response, enabling standard RT measurement. For the error-correcting response, evidence first accumulates to evoke an initial incorrect response and then continues to accumulate to later evokes an error-correcting response. This enables the measurement of RT2, the time from target presentation until the error-correcting response.

The hypothesis of cognitive continuity implies that the evidence accumulation process does not end once a response boundary is reached; rather, the evidence accumulation process continues and could lead to a reversal of the initial response^5,33^. Thus, the time from target presentation to a correct first response, or to an error-correcting response, would each reflect the time required for the evidence accumulation process to reach the correct response boundary. Consequently, in non-lava trials, we measured error-correcting responses as the time between the central rat’s color assignment and the initiation of the reversal of an incorrect first response. We name this measure RT2, which is intended to provide an additional measure of the time required for the evidence accumulation process to reach the correct response boundary. A simplified evidence accumulation process is illustrated in Fig.  2.

#### Error-correction in lava trials

To operationalize error-correction in lava trials, we assumed that go and stop responses are determined by a competition between two independent cognitive processes, as outlined by the independent race model^17,18^. From this perspective, the winner of a competition between a ‘go runner’ and a ‘stop runner’ determines whether a response is initiated or inhibited. Thus, stop-signal reaction time (SSRT), which is calculated in typical stop-signal tasks, is an estimate of the time it takes the stop runner to reach the competition’s ‘finish line’ and inhibit a response^17,18^.

**Fig. 3:**
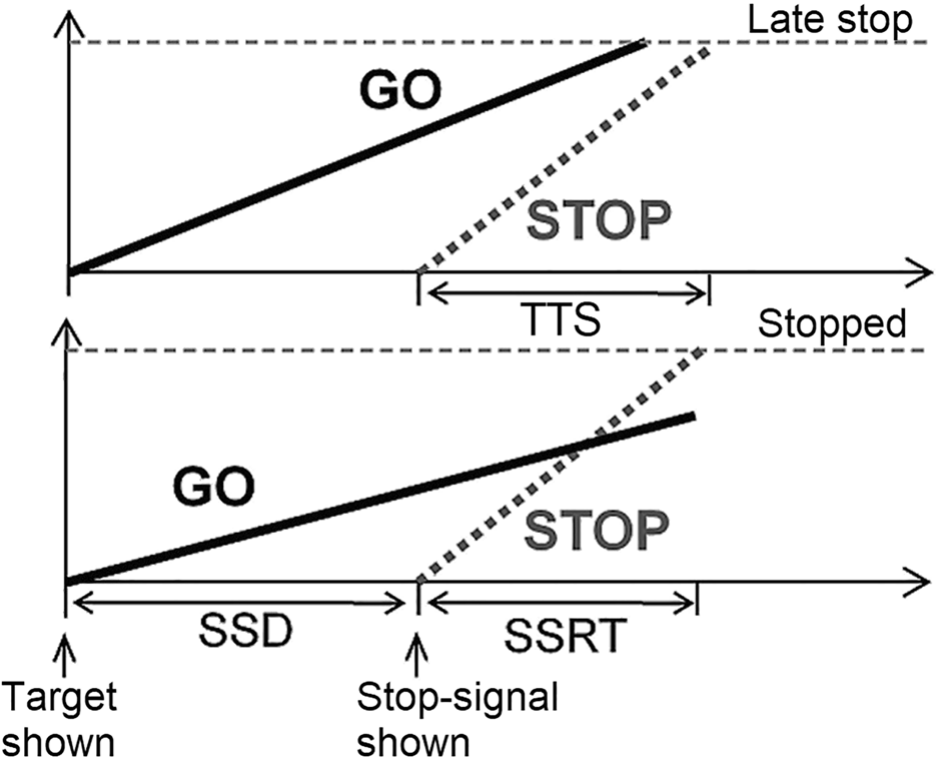
A competition between response initiation (go) and inhibition (stop) processes. In the top part, the go process reaches the finish line before the stop process, leading to an initial response that is later stopped. In the bottom part, the stop process reaches the finish line before the stop process. SSD, stop-signal delay, is the time between the target presentation and the stop-signal presentation. TTS, time-to-stop, is the time between the onset of the stop-signal and the late stop. SSRT is the stop-signal reaction time.

The hypothesis of cognitive continuity implies that the stop runner does not ‘quit’ once a go response is initiated and can reach the finish line in time to inhibit the ongoing response, as shown in Fig. 3. In Tunnel Runner’s lava trials, the inhibition of an ongoing response occurs when a player stops pressing the initial response button. Thus, the time from the presentation of a stop-signal until the inhibition of the ongoing go response, which we call the time-to-stop (TTS), reflects the time it takes for the stop runner to reach the finish line in trials where the go runner made it first. This means that both TTS and SSRT measurements should reflect the time it takes for the stop runner to reach the finish line. However, since players may inhibit their responses for reasons other than the stop-signal, TTS measurements require response inhibition to be followed by a corrective response (such as pressing L to move back and away from the lava), showing clear recognition of the mistaken initial response. On average, players took 191 ms between the inhibition of the ongoing response and the initiation of the corrective response. Thus, TTS is measured as the time between the stop signal and the stopping of the ongoing initial response, which was then followed by a corrective response.

#### Error-correcting responses differ from correct first responses

Cognitive continuity does not imply that error-correcting responses are identical to correct initial responses, since error-correcting responses can only be observed when the evidence accumulation process initially favored an incorrect first response or the go-runner initially won the competition. In other words, error-correcting responses selectively reflect trials where the cognitive processes under investigation performed worse than when a correct first response was initiated. Thus, error-correcting responses should take longer to make than correct first responses in a manner that may differ between individuals. Furthermore, several studies suggest that while initial and error-correcting responses share much in common, there are discontinuities between the two^33,37,38^. As such, error-correcting responses are unlikely to be directly comparable to correct first responses, and their use requires appropriate statistical adjustments.

### Procedure

We used the data that we originally collected for validating Tunnel Runner^4^, which consisted of two online studies of Tunnel Runner. We kept the original two-studies structure to limit our researcher degree-of-freedom, and because it enabled us to independently replicate the results of our analyses. In the studies, before the informed consent form, participants completed a brief test to check whether Tunnel Runner could be displayed with 50 frames-per-second or more, ensuring good player experience and precise measurement^4^. Following consent, participants completed questionnaires and played Tunnel Runner.

### Sample description

We conducted two studies through CloudResearch^39^, recruiting CloudResearch-approved Mechanical Turk users from the USA with at least 95% approval rate on at least 1,000 human intelligence tasks. These criteria should ensure a high-quality participant pool^39^. Participants’ median age was 38 (interquartile range: 17) in study 1, and 38 (interquartile range: 13) in study 2. Of the 117 participants in study 1, 73 identified as men, and 99 reported gaming on a weekly basis. Of the 121 participants in study 2, 81 identified as men, and 101 reported gaming on a daily basis.

As recommended to ensure high data quality in Tunnel Runner^4^, we excluded data at both the trial and individual levels. At the player level, we used a scoring system^4^ in which specific response patterns incur one or two points, with two or more points leading to the exclusion of player data. The following criteria led to a player’s exclusion: average frames-per-second lower than 35 across the study (study 1: 2, study 2: 1); first response accuracy no higher than 3 standard errors from 0.5 (11, 18); and non-response on more than 10% of non-lava trials (7, 2). Whereas at least two of the following were sufficient for exclusion: stopping rate above 0.7 or below 0.3 in lava trials (11, 8); correcting mistaken first movements in less than 30% of opportunities in non-lava trials (18, 13); correcting first movements in less than 30% of opportunities in lava trials (11, 9); failing to respond in more than 3% of non-lava trials (18, 17); and average frames-per-second below 45 (2, 2). Failure to meet these criteria led to the loss of 22 players in study 1 and 31 in study 2.

We applied additional player-level filters separately to the analyses of the ice effect, flanker effect, and SSRT and TTS measures. Since we focused on the use of error-correction data, we only considered players who had at least 3 error-correcting responses per relevant trial type. This led to a loss of 15 and 11 players for studies 1 and 2’s flanker effects, a loss of 2 and 0 players for studies 1 and 2’s ice effects, and a loss of 1 and 2 players for studies 1 and 2’s SSRT. Furthermore, we only calculated SSRT for players whose rate of stopping on lava trials was neither higher than 70% nor lower than 30%, as required by the integration method^8,18^, losing 2 and 0 players per studies 1 and 2’s SSRT and TTS measures.

Our analyses of ice and flanker effects on correct first response RT (RT1) excluded trials^4^ with RT1 lower than 300ms or higher than 1,500ms after the central rat’s color assignment or whose responses were more than 3 standard deviations from a players’ average RT1 per condition. We also excluded error-correcting responses that came more than 1 second after an initial response or a stop-signal or were further than 3 standard deviations from a player’s average error-correction time per condition. We did not apply trial-level filtering to the calculation of SSRTs^18^. The average number of trials analyzed per participant, measurement type, and study are described in Table 1.

### Data analytic approach

All statistical tests were two-sided with a $\alpha$ of .05 and were accompanied by 95% confidence intervals. We fitted hierarchical regression models using R package lme4^40^ and tested the models’ fixed effects with cluster-robust standard errors of type 2^41^ from the ClubSandwich package^42^ to mitigate heteroscedasticity. We used hierarchical regressions to account for the clustering of responses at the level of an individual participant and estimated individual differences in responses to cognitive challenges via the corresponding random slopes or intercepts obtained from the regression models. Hierarchical models, particularly joint models, are highly effective in estimating individual differences and their associations in the presence of measurement error^30,43–45^. When testing the models’ fixed terms, we assessed the normality of the models’ residuals and random effects with QQ-plots. Hierarchical regression models are robust against non-normality^46^, such that only very severe non-normality would have required us to change the analyses.

Hierarchical regression models allow cognitive measurements to reflect theoretical assumptions^30^, making these models suitable for embodying the hypothesis of cognitive continuity when jointly modeling the time from target presentation to correct first responses (RT1), and from target presentation to an error-correcting response (RT2). We modeled and measured individual differences in ice and flanker effects with fixed effects that accounted for trial type, response type, and for their interaction. Crucially, the models’ random effects accounted for individual-level variability per trial type and response type but not for their interaction. This random effect structure embodied the assumption that individual differences in the effect of trial type (the conflict effect) are shared between response types.

Since we calculated players’ SSRTs separately per matching and mismatching flanker trials using the integration method^17,18^, hierarchical models could not model players’ SSRT alongside their time-to-stop (TTS) measures. Where TTS reflects the time between a stop-signal and the stopping of an ongoing incorrect response. Previous work on Tunnel Runner^4^ showed that its SSRT can be validly and reliably estimated as an average of two z-transformed SSRTs calculated separately in matching and mismatching flanker trials. Thus, when using players’ TTS to measure SSRT, we first z-transformed TTS and then averaged it with the two z-transformed SSRT sub-measures, thereby using TTS as a third SSRT sub-measure.

We estimated measurement reliability via McDonald’s $\omega$^47^ for SSRTs, and with the even-odd split-half method^48^ for the other measures. McDonald’s $\omega$ is a natural fit for SSRT, since^49^ it is calculated as the average of several z-transformed measures: two SSRT sub-measures calculated separately per matching and mismatching flanker trials, and also TTS when applicable. Split-half reliability is a natural fit for measures based on hierarchical regression, as it is often used with cognitive measurements and would penalize the model-based estimates for overfitting.

To estimate the uncertainty around reliability estimates, we used the recommended bias-corrected and accelerated (BCa) bootstrapping^49,50^ with 100,000 iterations. However, this procedure has not been established for calculating differences between the reliability of nested measurements, such as SSRT calculated with or without TTS, or flanker effect calculated with or without RT2. We used simulations to assess the validity of bootstrapping in this context of nested measurements, and we found that bootstrapping incorrectly estimates the correlation between the nested measurements’ reliability , and therefore incorrectly estimates the uncertainty around their differences. For this reason, we will not report confidence intervals around reliability differences and we will not generalize these differences beyond our specific samples and cognitive measurements.

To estimate and compare the potential of measurements based on error-correcting responses and initial responses, we needed to separate the number of trials used per measurement from the measurement’s ability to assess individual differences. For measurements based on hierarchical modeling, this was achieved with the precision statistic $\eta$^31^, which is the ratio between the standard deviations of individual differences and the residual standard deviation estimated by a statistical model. Since we calculated the two first-response-based SSRT sub-measures via the integration method, their precision could not be estimated in a comparable way.

### Statistical expectations

We aimed to assess cognitive continuity between conflict-based measures based on correct first responses and error-correcting responses, and to evaluate the measurement potential of error-correcting responses alongside their ability to supplement measures based on first responses. The assessment of cognitive continuity was driven by statistical expectations, and shaped our expectations regarding the ability of error-correcting responses to supplement initial response data. This, in turn, reflected back on our conclusions regarding cognitive continuity.

To establish cognitive continuity between conflict-based measures of different response types, we expected error-correcting responses to replicate the conflict effects seen in Tunnel Runner’s correct first responses^4^. These include the flanker effects of increased time until correct initial responses (RT1) and stop-signal reaction time (SSRT), and the ice effect of increased RT1. Furthermore, if conflict-based measures of error-correcting responses are cognitively continuous with conflict-based measures of correct first responses, then the two measurement types should strongly correlate with each other. This means that flanker effects on RT1 and time until error-correcting responses (RT2) should correlate, ice effects on RT1 and RT2 should correlate, and players’ SSRT and time-to-stop (TTS) should correlate. Thus, we expected players’ error-correcting responses to show ice and flanker effects on RT2 whose magnitudes were comparable to those observed on RT1. Furthermore, we expected players’ TTS to show a flanker effect comparable to the one observed on SSRT. If these expectations were met and error-correction measurements strongly correlated with first-response-based measurements, we concluded that cognitive continuity likely occurred between the two response types. Furthermore, whenever cognitive continuity likely held, we expected error-correcting responses to beneficially supplement first-response data. This would provide converging evidence regarding continuity between correct initial and error-correcting responses, as supplementing initial response data with dissimilar data should reduce reliability.

## Results

### Response tendencies

The prevalence of players’ error-correcting responses, described in Table 1, shows that error-correcting responses were common in Tunnel Runner, and were particularly common in lava trials. As most lava trials with an initial response were followed by a delayed stop followed by a correction, which allowed the measurement of TTS. Players’ mean RTs per trial and response types can be found in the Supplementary material.

**Table 1:** Average number of trials per participant per measurement type across the studies. SSRT refers to stop-signal reaction time.

Measurement type	Study 1: trials per participant	Study 2: trials per participant
Ice effect: correct first responses	242.1	242.6
Ice effect: error-corrections	62.2	69.4
Flanker effect: correct first responses	193.9	191.5
Flanker effect: error-corrections	28.5	32.6
SSRT: lava trials	84.0	84.0
Time-to-stop in lava trials	28.8	35.8

### Do error-correcting responses show cognitive continuity with initial responses in conflict-based cognitive measurements?

**Table 2:** Conflict effects on error-correcting responses and their comparisons with conflict effects on first responses.

Source	Study 1: mean (95% CI)	Study 2: mean (95% CI)
Flanker effect on RT2	44.2* (26.1–62.3)	58.7* (41.9–75.6)
Difference between flanker effects on RT2 and RT1	−11.7 (−30.2–6.7)	−4.9 (−21.6–11.9)
Ice effect on RT2	49.7* (35.2 − 64.2)	48.6* (34.5 − 62.8)
Difference between ice effects on RT2 and RT1	−35.0* (−57.8–−12.2)	−39.9* (−62.7–−17.1)
Flanker effect on TTS	15.2* (8.8–21.7)	15.5* (8.2–22.7)
Difference between flanker effects on TTS and SSRT	−1.9 (−12.4–8.6)	−4.4 (−14.4–5.7)

RT1 is the time to correct first responses, RT2 is the time from color assignment until error-correcting responses, SSRT is stop-signal reaction time, and TTS is the time between the stop-signal and the stopping of an initial response that is followed by a corrective response. Confidence intervals and significance levels were based on hierarchical regression models for all but the last row, which is based on paired-sample t-tests. All measurements are at the millisecond unit. * Significant difference.

**Fig. 4:**
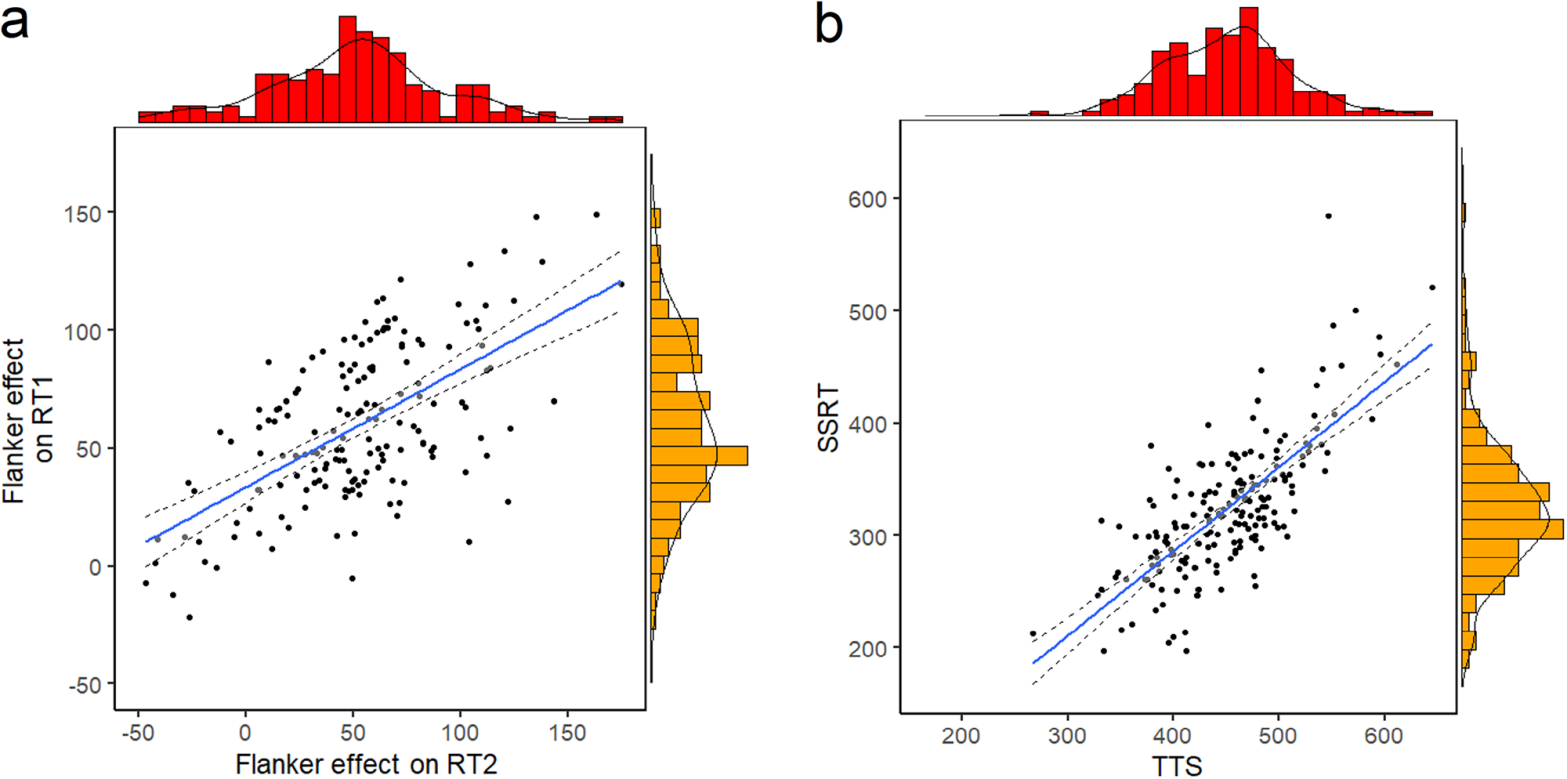
Correlations between first response and error-correction measurements. (**a**): The correlation between players’ flanker effects on correct first responses (RT1) and the time from color assignment until error-correcting responses (RT2) across both studies. (**b**): The correlation between players’ stop-signal reaction time (SSRT − transformed to the distribution of SSRTs in matching flanker trials) and the time between the stop-signal and the stopping of an initial response that is followed by a corrective response (TTS) across both studies. Dashed lines reflect the standard error around an estimate; all measurements are at the millisecond unit.

Given the assumption that players’ error-correcting responses reflect a continuation of the same cognitive functions measured with the ice and flanker effects, we expected the times from the central rat’s color assignment until correct initial responses (RT1) and from the central rat’s color assignment until error-correcting responses (RT2) to show comparable conflict effects. Furthermore, we expected conflict effects on RT1 to strongly correlate with conflict effects on RT2. To assess these expectations, we used joint hierarchical regression models with maximal random effect specification^51^. With random and fixed terms for the intercept, trial type, response type, and the interaction between response type and trial type.

As described in Table 2, hierarchical regression models showed significant flanker effects on RT2 in studies 1 (m = 44.2 ms, $\textit{t}(77.7)$ = 4.77, *p* < 0.001) and 2 (m = 58.7 ms, $\textit{t}(74.6)$ = 6.82, *p* < 0.001) which were not significantly different from (though quantitatively shorter than) flanker effects on RT1 in both studies 1 (m = −11.7 ms, $\textit{t}(78.3)$ = −1.25, *p* = 0.216) and 2 (m =−4.9 ms, $\textit{t}(74.7)$ = −0.57, *p* = 0.570). Furthermore, flanker effects on RT1 and RT2 strongly correlated in both studies 1 (*r* = 0.53, 95% CI: 0.35–0.66, $\textit{t}(83)$ = 5.62, *p* < 0.001) and 2 (*r* = 0.63, 95% CI: 0.48–0.75, $\textit{t}(78)$ = 7.22, *p* < 0.001), as shown in Fig. 4. These results suggested that the flanker effects on RT2 reflected a continuation of the cognitive processes measured by the game’s flanker effects on RT1.

To better understand how the flanker effect influenced RT2, we separated the flanker effect on RT2 into its constituent effects on the time it took participants to make mistaken initial responses and on the time from incorrect initial responses to error-correcting responses. Using hierarchical regression models with random and fixed terms for the intercept and trial type, we found significant flanker effects on the time to incorrect initial responses in both studies 1 (m = 39.4 ms, 95% CI: 23.9–55.0, $\textit{t}(71.9)$ = 4.97, *p* < .001) and 2 (m = 44.9 ms, 95% CI: 30.6–59.2, $\textit{t}(69.7)$ = 5.15, *p* < .001). Furthermore, we found an inconsistent flanker effect after the incorrect initial response, which significantly increased time from incorrect initial responses to error-correcting responses in mismatching trials in study 2 (m = 14.3 ms, 95% CI: 3.2–25.2, $\textit{t}(69.7)$ = 2.56, *p* = .013), though not in study 1 (m = 9.0 ms, 95% CI: −2.0–19.9, $\textit{t}(68.6)$ = 1.60, *p* = 0.114).

As described in Table 2, we found ice effects on RT2 in both studies 1 (m = 49.7 ms, $\textit{t}(92.4)$ = 6.73, *p* < 0.001) and 2 (m = 48.6 ms, $\textit{t}(87.1)$ = 6.73, *p* < 0.001), although the effects on RT2 were considerably shorter than the effects on RT1 in studies 1 (m = −35.0 ms, $\textit{t}(96.3)$ = −3.01, *p* = 0.003) and 2 (m = −39.9 ms, $\textit{t}(89.6)$ = −3.44, *p* <.001). Furthermore, ice effects on RT1 and RT2 showed a non-significant (though quantitatively negative) correlation in study 1 (*r* = -.15, 95% CI: −0.34–0.04, $\textit{t}(96)$ = −1.48, *p* = 0.141), and a significant negative correlation in study 2 (*r* = −0.39, 95% CI: −0.55–0.20, $\textit{t}(89)$ = −3.99, *p* < 0.001), precluding a strong positive correlation between the measures. These results led us to conclude that the ice effects on RT2 likely did not reflect a continuation of the cognitive processes measured by the ice effects on RT1.

Given our assumption that players’ late stopping responses reflect a continuation of the same cognitive functions responsible for their stop-signal reaction times (SSRTs), we expected players’ time-to-stop (TTS), calculated as the time from the stop-signal until the inhibition of an ongoing initial response, to show a flanker effect comparable to the one observed on SSRT. Furthermore, we expected SSRT and TTS measures to strongly correlate. To assess these expectations, we calculated SSRTs via the integration method, and players’ TTS via hierarchical regression models with fixed and random terms for intercept and trial type.

As shown in Table 2, we found flanker effects on players’ TTS in both studies 1 (m = 15.2 ms, $\textit{t}(90.4)$ = 4.61, *p* < 0.001) and 2 (m = 15.5 ms, $\textit{t}(87)$ = 4.2, *p* < 0.001), which paired-samples t-tests showed were not significantly different from (though quantitatively shorter than) the flanker effects on SSRT in studies 1 (m = −2.6 ms, $\textit{t}(96)$ = −0.46, *p* = 0.628) and 2 (m = −4.4 ms, $\textit{t}(88)$ = −0.88, *p* = 0.382). Furthermore, as shown in Fig. 4, players’ SSRT scores strongly correlated with their TTS scores in both studies 1 (*r* = 0.76, 95% CI: 0.65–0.83, $\textit{t}(95)$ = 11.24, *p* < 0.001) and 2 (*r* = 0.74, 95% CI: 0.63–0.82, $\textit{t}(87)$ = 10.39, *p* < 0.001). These results led us to conclude that players’ TTS likely reflected a continuation of the cognitive processes measured by their SSRT.

### Can error-correcting responses be used alongside initial responses to improve the reliability of conflict-based cognitive measurements?

**Table 3:** Reliability of measurements based on first responses only, and on first responses combined with error-correcting responses.

Measurement type	Reliability: first responses only (95% CI)	Reliability: first responses and error-corrections (95% CI)
Study 1: flanker effect	0.732 (0.605–0.805)	0.754 (0.624–0.827)
Study 2: flanker effect	0.771 (0.619–0.850)	0.779 (0.624–0.857)
Study 1: ice effect	0.874 (0.810–0.912)	0.847 (0.744–0.902)
Study 2: ice effect	0.814 (0.718–0.869)	0.802 (0.686–0.864)
Study 1: SSRT	0.831 (0.727–0.892)	0.877 (0.818–0.908)
Study 2: SSRT	0.847 (0.728–0.898)	0.879 (0.797–0.918)

SSRT is stop-signal reaction time. Reliability was estimated via odd-even split-halves for the ice and flanker effects and via McDonald’s $\omega$ for SSRT. Confidence intervals were estimated via bias-corrected accelerated bootstrapping with 100,000 iterations.

If error-correcting responses are indeed continuous with correct initial responses, then they should be able to enhance the psychometric properties of the measurements. Thus, we supplemented first-response-based measurements with error-correcting responses in two ways. For ice and flanker effects, we used hierarchical regression models with fixed and random effects of trial type and response type and only a fixed effect for their interaction. This model structure embodied the assumption that individual differences in conflict effects were shared between response types. For SSRT calculations, we z-transformed players’ TTS as estimated by hierarchical regression with fixed and random intercept and only a fixed trial type effect. We then averaged players’ z-transformed TTS alongside their z-transformed SSRTs calculated separately per matching and mismatching flanker trials.

As shown in Table 3, supplementing first-response-based measurements with error-correcting responses resulted in quantitatively modest increments to our measurements’ reliability that ranged from .008 to .046 for flanker effect and SSRT measurements. While we cannot generalize these results beyond these samples, they are in-line with the expectation of continuity between initial correct and error-correcting responses for these measures. The SSRT and flanker effect estimated via first responses were nearly identical to those obtained by combining first response and error-correction data (*r*s = 0.97). In contrast, quantitatively modest reliability reductions of −0.027 and −0.012 were seen in studies 1 and 2’s measures of the ice effects, and the two measurement approaches were not as strongly correlated (study 1: *r* = 0.86; study 2: *r* = 0.93) as before. This provides further support for discontinuity between the ice effects.

Since it is more difficult to improve the reliability of measurements with high initial reliability, the observed reliability increments require further elaboration. Using formula 3 from Kucina et al. ^31^ we translated our observed reliability increments into a number of additional trials and then calculated how this amount of trials would improve a typical^31^ reliability of 0.50. Crucially, the results of this procedure depend only on the initial reliability and how it changes. Following this procedure, we found that increments to the flanker effect’s reliability reflect as many additional trials as increments from 0.50 to 0.576 in study 1, and to 0.540 in study 2. Whereas increments to the SSRT’s reliability reflect as many additional trials as increments from 0.50 to 0.767 in study 1, and to 0.738 in study 2. These results illustrate the potential reliability benefits of cognitively-continuous error-correction data.

### Can error-correcting responses be used as cognitive measurements on their own?

**Table 4:** Precision of the different measurements, defined as the ratio between the standard deviation of individual differences and of the measurement noise estimated by a statistical model.

Measurement	Study 1: $\eta$	Study 2: $\eta$
Flanker effect on RT1	0.24	0.27
Flanker effect on RT2	0.24	0.24
Ice effect on RT1	0.50	0.44
Ice effect on RT2	0.32	0.32
TTS	0.73	0.66

RT1 is the time to correct first responses, RT2 is the time to error-correcting responses, SSRT is stop-signal reaction time, and TTS is time-to-stop.

To assess the potential of error-correcting responses as separate cognitive measurements, we estimated individual differences and measurement noise using hierarchical regressions that considered first response data separately from error-correction data. For the ice and flanker effects on RT1 and RT2, the models contained fixed and random terms for intercept and trial type. For the TTS, the models included fixed and random intercepts, and only a fixed trial type term since individual differences due to trial type (the flanker effect on TTS) were minimal.

As shown in Table 4, RT2 measures of flanker and ice effects were no more precise than RT1 measures. This means that the game’s RT2 conflict effect measures, similarly to the RT1 conflict effect measures, require hundreds of response trials to achieve acceptable measurement reliability on their own. Consequently, it is not feasible to use these RT2 conflict effect measures on their own. In contrast, the TTS measure showed very high precision, sufficient to achieve excellent split-half reliability of 0.924 and 0.936 in studies 1 and 2. Which suggests that TTS can serve as a standalone measurement.

## Discussion

We used behavioral data collected from Tunnel Runner to address key theoretical and psychometric challenges to the use of error-correcting responses in conflict-based cognitive assessment. Specifically, we assessed whether cognitive continuity held between first responses and error-correcting responses in the game’s conflict-based measurements, and we examined how error-correcting responses can be combined with initial responses, and whether error-correcting responses can measure cognitive functions on their own. Our results supported cognitive continuity between initial and error-correcting responses in the game’s measurements of interference control and response inhibition but not for response-rule switching. Furthermore, supplementing first-response data with error-correcting responses quantitatively increased the reliability of the flanker effect and SSRT measurements, and decreased the reliability of the ice effect measurement. Lastly, error-correcting responses showed the ability to measure response inhibition, via TTS, separately from first responses, which was not the case for interference control and response-rule switching. Overall, our results suggest that cognitive continuity between initial and error-correcting responses can extend to conflict effects, although this is not always the case. This key finding suggests that error-correcting responses can enhance or, in the case of response inhibition, even replace first-response-based conflict measures.

### Explanation

We found cognitive continuity between first-response-based and error-correction-based measures of response inhibition and interference control, yet not for response-rule switching. The ice effects on error-correcting responses were nearly half the size of the effects on correct responses, and the ice effects on the two response types did not positively correlate. We speculate that this discontinuity can be explained in reference to neurophysiological indications that response-rule switching involves the initial enhancement of effortful control mechanisms that is later followed by reduced action monitoring^36^, and that the reconfiguration of response rules is delayed after task switching compared to other conditions^52^. Specifically, if delayed reduction in action monitoring translates into delayed reduction in response caution in ice trials, then less evidence would need to accumulate to initiate error-correcting responses compared to initial responses. Furthermore, delayed reconfiguration of response rules could mean that the evidence accumulation rate is increased after initial responses, as re-configuration was more likely to have already occurred. These processes could cause correct responses to be easier to make later in ice trials, potentially explaining the weaker ice effects on error-correcting responses. Furthermore, if individual differences in the impact of these processes are unrelated to or negatively associated with the initial ice effect, then these processes could explain why the ice effects on RT1 and RT2 did not positively correlate.

The continuity between first-response-based and error-correction-based measures of response inhibition and interference control should be interpreted with caution. Our results do not imply that error-correcting responses are identical to correct first responses, nor do they suggest that the cognitive processes involved in error-correcting responses are completely unchanged compared to initial responses. Our continuity results are compatible with suggestions that response boundaries^37^ and evidence accumulation rates^38^ can differ between error-correcting responses and initial responses^33^. What our results suggest is that if there are differences between the cognitive processes involved in the two response types, then these differences exert a small cumulative influence on conflict-based measures of interference control and response inhibition.

When cognitive continuity was otherwise shown between response types, supplementing initial responses with error-correcting responses repeatedly resulted in quantitative improvements to reliability. This provided further converging evidence for continuity, as additional data of the same type should increase reliability^20^. In the model-based approach we used to measure interference control via the flanker effect, error-correcting responses were treated as additional trials for measurement. Psychometrically, this means that any added value of the error-correcting responses would depend on the number and precision of these responses and the measurement’s initial reliability^31^. Such that the value of cognitively-continuous error-correction data might increase with the number of error-correcting responses and their measurement precision, yet diminish as the measurement’s initial reliability increases^20,31^. Therefore, since the flanker effects showed acceptable initial measurement reliability and the number of error-correcting responses was not large, only quantitatively modest reliability increments could be observed. However, if a measurement has high precision yet low reliability because of a limited number of correct initial responses, then cognitively-continuous error-correcting responses, if common enough, could provide considerable reliability improvements.

The combination of first-response-based response inhibition measurements with error-correcting responses was achieved by averaging three distinct measurements and thus brings its own psychometric considerations. Specifically, the TTS measurement needs to be highly reliable on its own, enabling it to strongly correlate with the other two SSRT measurements to form an internally consistent set of distinct measurements. In addition, the TTS measure, unlike the other error-correction measures, showed the ability to reliably measure response inhibition on its own using achievable amounts of error-correcting responses. This high reliability was driven by the measurement’s high precision, which was likely achieved because the TTS measure was not a difference score^20,21^.

### Implications for theories of cognitition

While error-correcting behaviors are a common aspect of daily lives, they received limited theoretical attention in the domain of cognitive control, where the emphasis has been on highlighting continuities and discontinuities with initial responses^33^. Recent theoretical perspectives on cognitive control, such as Gated Cascade Diffusion^53^, and Binding and Retrieval in Action Control (BRAC)^54^, seek to explain the nature of initial responses, and how error corrections manifest earlier in the trial^53^ or in subsequent trials^54^. In particular, BRAC attempts to provide a direct explanation for various conflict effects and the relationship between performance in earlier and later trials, including post-error slowing and congruency sequence effects. However, it is difficult to apply these theoretical perspectives to error-correcting responses that follow mistaken initial responses in the same trial, as these perspectives neither attempt to explain nor are based on these types of error-correcting responses. This is unfortunate because individuals have a natural tendency to correct errors, which manifests in cognitive tasks even when error-corrections are not allowed^5^ and plays a role in shaping responses in subsequent trials^55,56^. Thus, error-correcting responses can help in understanding and linking between initial responses and trial sequence effects; crucially, error-correcting responses are worthy of consideration on their own and could play an important role in further understanding human cognition.

By examining cognitive continuity between conflict effects on initial and error-correcting responses, our results open a new path towards integrating error-correcting responses into theories of human cognition. Our results also showcase Tunnel Runner’s unique ability to evoke and measure error-correcting responses, and position it as a powerful tool for the study of error-correcting responses. This should help theories of cognition advance toward a better understanding of the dynamic nature of human cognition.

### Implications for cognitive measurement

Typical conflict-based cognitive assessment tools face criticism for being boring^1,4^ and behaviorally restrictive^3,4^ while achieving limited psychometric reliability^16^. Cognitive games such as Tunnel Runner, which create a more fluid, dynamic, and engaging cognitive assessment experience^4^, can mitigate some of these issues while creating new challenges and opportunities. Since cognitive games tend to evoke a higher prevalence of incorrect first responses^4,13^, they lose more correct RT data and, therefore, show reduced efficiency. However, by enabling and using error-correcting responses, cognitive games may be able to compensate for much of, or even potentially exceed, the lost information. Furthermore, error-correcting responses can create new opportunities for data collection, as was the case with the time-to-stop measure. A similar measure could be implemented in other response inhibition paradigms, such as go/no-go, to efficiently provide an additional yet distinct^57^ way to measure response inhibition. Alternatively, time-to-stop could help assess theoretical constructs beyond SSRTs, such as failures to initiate the stop runner^58^.

The use of error-correction data could complement other approaches for improving the reliability of cognitive assessment. Increasing the difficulty of conflict tasks can enhance their measurement reliability^31^, although it may lead to more incorrect initial responses and thus greater data loss^4^. By considering error-correcting responses, this data loss can be minimized. Furthermore, the use of error-correcting responses requires careful use of hierarchical models, making it a natural fit for theoretically-informed hierarchical models of response data, which were shown to enhance reliability^30^. Overall, our results should encourage the allowance of error-correction in cognitive assessment and promote the use of the measurement opportunities created by error-correcting responses. This should help cognitive assessment tools better capture the dynamic nature of human cognition while providing participants with an improved experience^4^.

### Implications for other types of behavioral measurements

Differences in initial responses to different types of stimuli are used in various fields and for different purposes, such as attitude assessment in consumer research and user modeling in human-computer interaction. Although these application areas often allow users to change their minds and reverse an initial decision they perceive as incorrect. Since there is no reason to expect continuity to be restricted only to cognitive measurements, our results imply that actions that reverse an initial decision (or change-of-mind data^33,37^) could be useful for purposes other than cognitive assessment. Thus, our results and the approach we took in defining, measuring, and utilizing change-of-mind responses and establishing continuity between different response types pave a path for the use of change of mind data in various research and application areas.

### Limitations and future directions

Our studies contain several limitations, which we outline here. First, the number of players whose in-game responses could not be analyzed was high, although in line with online cognitive games studies^4,13,31^. Second, we did not assess the impact of using error-correcting responses on test-retest reliability nor on the prediction of other variables. We emphasize the structure of error-correcting responses and their relationship with initial responses, leaving the prediction of other measurements for future work. Third, our samples consisted mainly of gamers, meaning that our results might not generalize to non-gamers. This is a consequence of the self-selecting nature of online sampling and Tunnel Runner’s requirement for a functional graphical processing unit in a player’s computer. Fourth, we did not model error-correcting responses with evidence accumulation models, as these are not often used to assess individual differences^7,59^. Nevertheless, evidence accumulation models can be made to fit and explain error-correction data and could benefit from additional data^5,7^. Fifth, we did not consider error-correcting responses in relation to conflict effects on accuracy scores. Although accuracy levels are an integral part of many conflict-based measurements^60^, we did not find a good way to conceptualize the use of error-correcting responses for accuracy measures. Lastly, we did not model in-game task-switching costs, which could add noise to our continuity results. An approach to modeling task-switching with Tunnel Runner’s multiple elements would be complex and first needs to be established. Thus, future work could consider the impact of error-correcting responses on test-retest reliability, assess cognitive continuity using different behavioral measurements, and/or develop better methods to use error-correcting responses for cognitive assessment, including accuracy scores, potentially via computational modeling that account for trial-switching costs, or via time-to-event analyses.

### Conclusions

We demonstrated that error-correcting responses can be cognitively continuous with initial responses for conflict-based measurements of interference control and response inhibition, such that error-correcting responses can supplement or even replace first-response-based cognitive measurements. However, cognitive discontinuity can also apply in some conflict-based measurements, as was the case for response-rule switching, in which case error-correcting responses should not be used alongside or instead of initial responses. These results improve our understanding of the dynamics of human cognition that go beyond initial responses, call further theoretical attention toward error-correcting responses, and pave a path toward the use of change of mind data for cognitive assessment and other research and application areas.

## Supplementary Information

Supplementary Information.

## Data Availability

Data and scripts are available as Supplementary material and at https://osf.io/qjhwv/. Questions should be addressed to the first author. A public demo of the game is available at https://tunnel-runner.itch.io/tunnel-runner-demo.
